# The Optimal Cut-Off Value of Blood Stasis Syndrome Score in BSS Diagnosis in Korea

**DOI:** 10.1155/2017/8049481

**Published:** 2017-09-11

**Authors:** Byoung-Kab Kang, Tae-Yong Park, Jeeyoun Jung, Mimi Ko, Myeong Soo Lee, Ju Ah Lee

**Affiliations:** ^1^KM Fundamental Research Division, Korea Institute of Oriental Medicine, Daejeon, Republic of Korea; ^2^Institute for Integrative Medicine, Catholic Kwandong University International St. Mary's Hospital, Incheon 22711, Republic of Korea; ^3^Clinical Research Division, Korea Institute of Oriental Medicine, Daejeon, Republic of Korea

## Abstract

**Objective:**

In the traditional oriental medicine, it is sometimes difficult to diagnose Blood Stasis Syndrome (BSS) in patients, because the diagnosis of BSS is based on the subjective signs and symptoms of patients. This study is aimed at developing the prediction tool of BSS using cut-off value for BSS score. The identification of a cut-off value for BSS score to diagnose BSS would be helpful.

**Methods:**

A total of 887 patients admitted to six traditional Korean medical hospitals in 2013 and three hospitals in 2014. All patients have an identical pattern as a result of diagnostic decision of two experts. The cut-off value for BSS score for BSS diagnosis was determined by the receiver-operating characteristic curve.

**Results:**

The area under the curve of this curve was 0.897. The optimal cut-off point for detection of BSS was 49.0. The sensitivity and specificity of this cut-off value were 80.8% and 83.2% in modelling data (2013 dataset) and 84.6% and 83.1% in validation data (2014 dataset), respectively.

**Conclusion:**

Our study suggests that a BSS score cut-off value of 49.0 can be used to detect BSS in the traditional Korean medical hospitals. This cut-off value for diagnosis of BSS will make up the lack of objectivity.

## 1. Introduction

Pattern identification (PI) is also called Bian Zheng (辨症), syndrome differentiation, pattern diagnosis, or pattern classification. The World Health Organization (WHO) defines PI as “diagnosis of the pattern, through comprehensive analysis of symptoms and signs, which has implications for determining the cause, nature and location of the illness and the patient's physical condition” [1].

Blood Stasis Syndrome (BSS) is one of the most common PI and is related to a variety of diseases. BSS is one of the major syndromes in Traditional East Asia medicine. BSS, also called Eohyul syndrome in Korean term and Oketsu Syndrome in Japanese term, refers to the fact that the circulation of blood is not smooth or bold flow is stagnant and forms stasis [2]. BSS is the compound of various manifestations including pain that occurs in a fixed location, dark-purple face or tongue, bleeding, blood spots under the skin, and an astringent pulse among other features [2].

The development of diagnostic criteria for BSS has been performed from 1982 to 2011. The diagnostic criteria for BSS consists of macroscopic items (symptoms and signs) and microscopic items (indicators of physical or chemical test) [3]. The diagnostic criteria for BSS (1986) was the most commonly used and highest cited one in China [4].

For the last 30 years, the diagnostic criterion for BSS was mainly established by the Committee Consensus [4–11]. However, the symptoms and signs combination based on diagnostic criterion for the draft Coronary Heart Disease- (CHD-) BSS in China [12]. According to the diagnostic criterion for BSS (1986) or the draft CHD-BSS diagnostic criterion, the BSS diagnosis indicated good reliability of the new criterion with sensitivity of 94.36% and specificity of 89.38% [3].

In several previous studies, the main signs and symptoms determined by clinical literature or experience of experts have been used as the basis for differentiation in diagnosing BSS. “Diagnostic criteria for Oketsu” proposed by Terasawa et al. (1983) was used in Japan [10], but the validity of the content was not verified and the weighting criterion was not clear. Therefore, there is a need to develop a reliable diagnostic tool for BSS. Wang and Chen developed criteria for diagnosis of BSS using statistical analysis based on clinical experience in China [11], Yang et al. made a questionnaire by selecting 14 items with high importance for determining BSS in Korea [13].

Because only a diagnosis criterion of BSS could be made by doctor's subjective decision on the basis of the patient's symptoms and signs, suggesting a cut-off value for diagnosing BSS would be useful. However, there is currently no cut-off value available in Korea. The prompt and accurate treatment is necessary to obtain a better prognosis.

The purpose of this study is to identify the optimal cut-off value of the BSS score and use it to diagnose the BSS. Developing a BSS prediction tool using cut-off value for BSS score will be useful in diagnosing the BSS. Therefore, we carried out an analysis to determine the optimal cut-off value of score for diagnosing BSS.

## 2. Materials and Methods

### 2.1. Study Design

This study protocol was approved by IRB of the Korea Institute of Oriental Medicine (KIOM) and each traditional Korean medical hospital's ethics committee. This study was a community-based, multicentre trial adopting a cross-sectional observational design [14].

In 2013, patients admitted to the six traditional Korean medical hospitals: Kyung Hee Korean Medical Center, Kyung Hee University Korean Medical Hospital at Gangdong, Won Kwang Korean Medical Hospital, Jaseng Hospital of Korean Medicine, Cha Medical Center, and Pusan National University Korean Medicine Hospital and three traditional Korean medical hospitals: Gynaecology and Cardiovascular Department of Dongguk Korean Hospital at Ilsan and Jaseng Hospital of Korean Medicine in 2014.

All participants who submitted informed consent were recruited according to sex, age, and whether BSS or not at the same hospital. To determine the presumption of blood stasis/nonblood stasis the short-form questionnaire for blood stasis will be used and the participants will be divided into a blood stasis presumption group or nonblood stasis. The sort-form query for blood stasis consisted of possible items to check by participants and will be used only to divide the two presumption groups. The short-form questionnaire consisted of eight item-related signs or symptoms of BSS. Male participants who have four or more signs will be regarded as blood stasis presumption group; female participants who have five or more signs will be regarded as blood stasis presumption group [14].

### 2.2. Inclusion and Exclusion Criteria

All participants who submitted informed consent were recruited. The participants who meet all of the following requirements will be eligible for enrolment. The eligibility criteria will be as follows: males or females aged between 20 and 70 years who give their written informed consent to participate and agree to comply with the study regulations. The exclusion criteria are patients with any psychiatric condition that renders them unable to communicate, patients that are critically ill, pregnant women, or patients with any condition that could influence the study assessment.

### 2.3. Participants

Without discussion, two Korean medicine doctors completed the long-form questionnaire by assessing the participants independently in the same department within each site, before the expert diagnoses whether it is BSS or non-BSS. The experts had at least three years of clinical experience with BSS after finishing regular college education of traditional Korean medicine for six years. These assessments were conducted individually without discussion between the two experts in each site and made on the same day without delay, therefore minimizing the time difference between the former and the latter diagnoses. A total of 17 experts were involved in this study. All experts were well trained in standard operation procedures (SOPs). A total of 887 subjects from May 2013 to November 2014, who have an identical pattern as a result of diagnostic decision of two experts, were analyzed in this study.

### 2.4. Measurement

The BSS questionnaire had satisfactory reliability and validity using 33 items excluding 3 female items, although the BSS questionnaire consisted of 36 items [15]. The BSS score is calculated as the sum of 29 signs and symptoms on the 5-point scales, excluding a total seven items which were three items related to female, number of traffic accidents and operation, palpable abdominal masses (exchange from 5-point scales in 2013 to binary in 2014), and binary item (angina pectoris). The score of each item was calculated by the average of two experts.

### 2.5. Statistical Analysis

The two groups were compared on the basis of age, sex, body mass index (BMI), systolic blood pressure (SBP), diastolic blood pressure (DBP), and pulse rate. Continuous variables as age, SBP, DBP, and pulse rate are expressed as the mean ± SD and were compared using unpaired *t*-tests. Categorical variables such as sex and BMI are expressed as number and percentages and were compared using chi-square test or Fisher's exact test. The cut-off value for BSS score was determined by generating the receiver-operating characteristic (ROC) curve through logistic regression adjusted sex and age for the ability of the BSS score to detect BSS. The model was created using 2013 dataset and verified by 2014 dataset. Statistical significance was indicated by a value of *p* < 0.05. Statistical analysis was performed using SAS version 9.1.3 statistical software (SAS Institute, Cary, NC).

## 3. Results

### 3.1. General Characteristics of the Patients

The characteristics of both groups were compared in Table 1. The mean age of the non-BSS group (45.9 ± 12.7) is higher than the BSS group (44.2 ± 11.5). The percentage of female in the BSS group (68%) is higher than non-BSS group significantly. The mean score and standard deviation of each item of BSS and total BSS score are shown in Table 2.

### 3.2. The Sensitivity, Specificity, Positive Predictive Value, and Negative Predictive Value for Optimal Cut-Off Value of the BSS Score

**Figure 1:**
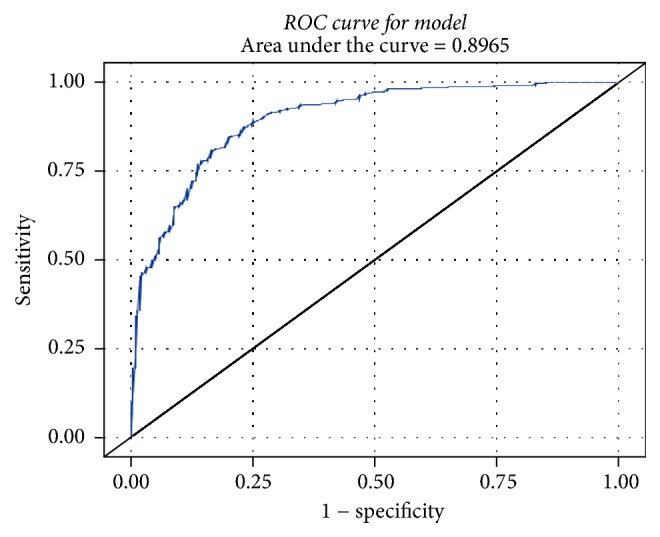
Area under the curve for ROC. ROC: receiver-operating characteristic.

We examined the sensitivity and specificity of various cut-off values of BSS score for predicting BSS from the ROC curve (Figure 1). The AUC of this curve was about 89.7%. We considered the BSS score with the shortest distance on the ROC curve as the optimal cut-off and determined that the optimal cut-off point to detect BSS was 49. The sensitivity and specificity of this cut-off were 80.8% and 83.2% in modelling data (2013 dataset) and 84.6% and 83.1% in validation data (2014 dataset), respectively. The sensitivity and specificity are useful for the diagnosis of BSS, when the BSS score is 49. The sensitivity, specificity, positive predictive value, and negative predictive value at various cut-off values of BSS score are also shown in Table 3.

## 4. Discussion

The present study aimed to identify the optimal cut-off value of the BSS score and use it to diagnose the BSS. We determined the optimal cut-off value for diagnosing BSS to be 49.0 score. The proposed value for diagnosing the BSS was based on the greatest discriminatory ability on ROC analysis. The BSS scores are useful for determining whether there is BSS or not. In this study, the BSS scores were much higher in the BSS group (55.7 (7.9), 57.7 (8.3)) than non-BSS group (48.8 (5.3), 43.7 (5.2)) in 2013 and 2014 dataset, respectively.

As shown in Table 3, the range of cut-off values 48–50 came out similarly as sensitivity, specificity, positive predictive value, and negative predictive value, although 49 was the cut-off value optimum. It can be helpful to diagnose BSS based on this criteria when assessing all the signs and symptoms of the participants. Generally, experts should diagnose BSS reflecting the overall items, but there are cases in which BSS is diagnosed with only a few items. Therefore, it is possible to relatively reduce the probability of performing different diagnoses when experts diagnose BSS for the same patient using the BSS cut-off value. The use of a cut-off value will help to standardize diagnosis of BSS.

It is imperative to determine accurate BSS score cut-off values in order to diagnose BSS in traditional Korean medicine. The diagnostic criterion for BSS (1986) or the draft CHD-BSS diagnostic criterion had good reliability of the new criterion with sensitivity of 94.36% and specificity of 89.38% [3]. The reason for the higher sensitivity and specificity of Chinese study than our results is that we are targeting various diseases, whereas Chinese study is only for patients with heart disease.

There were several limitations associated with this study. First, our study suggests that a BSS score cut-off value of >49.0 can be used to detect patients with BSS. However, our findings do not imply that one cannot use cut-off values other than >49.0. In this case, physicians may have to make a trade-off when choosing the cut-off value. A cut-off value of >49.0 could be chosen if experts are ready to accept more false positives. Second, it is cut-off point in patients with BSS in several disease. So the cut-off value specific for the each disease is needed and the sample sizes of each disease in this study were small. Finally, in the BSS or non-BSS presumption group, age and gender were not matched for specific diseases, especially gynaecological diseases.

In conclusion, we determined the optimal cut-off value for BSS score so that diagnosis score becomes 49.0. The area under the receiver-operating curve was 89.7%, and the sensitivity and specificity were 80.8% and 83.2% in modelling and 84.6% and 83.1% in validation, respectively. These results suggest that optimal cut-off value of the BSS score can be used in traditional Korean hospitals to diagnose BSS.

## Figures and Tables

**Table 1:** The characteristics of the patients with and without Blood Stasis Syndrome.

	2013		2014		Total		*P* value
	BSS (*N* = 250)	Non-BSS (*N* = 226)	BSS (*N* = 175)	Non-BSS (*N* = 236)	BSS (*N* = 425)	Non-BSS (*N* = 462)	
Sex							
Male	99 (39.6)	123 (54.4)	37 (21.1)	100 (42.4)	136 (32.0)	223 (48.3)	<0.0001
Female	151 (60.4)	103 (45.6)	138 (78.9)	136 (57.6)	289 (68.0)	239 (51.7)	
Age (year)	44.4 ± 10.9	44.6 ± 11.7	44.0 ± 12.3	47.2 ± 13.5	44.2 ± 11.5	45.9 ± 12.7	0.0368
BMI (kg/m^2^)							
Low (<20)	35 (14.0)	25 (11.1)	30 (17.1)	37 (15.7)	65 (15.29)	62 (13.4)	0.6684
Normal (20–24)	138 (55.2)	124 (54.9)	97 (55.4)	147 (62.3)	235 (55.3)	271 (58.7)	
Overweight (25–29)	65 (26.0)	68 (30.1)	40 (22.9)	44 (18.6)	105 (24.7)	112 (24.2)	
Obesity (≥30)	12 (4.8)	9 (4.0)	8 (4.6)	8 (3.4)	20 (4.7)	17 (3.7)	
SBP (mmHg)	119.4 ± 15.2	119.9 ± 15.5	119.8 ± 16.1	120.0 ± 14.0	119.2 ± 15.1	120.3 ± 15.0	0.2774
DBP (mmHg)	74.8 ± 11.1	75.3 ± 11.0	76.7 ± 11.9	75.9 ± 10.6	75.3 ± 11.2	76.0 ± 11.0	0.3797
Pulse rate (BPM)	74.6 ± 10.0	73.5 ± 10.4	76.7 ± 10.7	75.6 ± 10.7	75.5 ± 10.2	74.6 ± 10.8	0.2283

BMI: body mass index, SBP: systolic blood pressure, and DBP: diastolic blood pressure.

**Table 2:** Descriptive statistics of signs and symptoms of BSS and BSS score.

	2013		2014	
	BSS (*N* = 250)	Non-BSS (*N* = 226)	BSS (*N* = 175)	Non-BSS (*N* = 236)
Signs and symptoms of BSS				
(1) Having dizziness	2 (1)	1.5 (0.8)	2.1 (1.1)	1.4 (0.7)
(2) Having angina pectoris	—	—	—	—
(3) Having chest pain without angina pectoris	1.4 (0.7)	1.3 (0.5)	1.5 (0.9)	1.2 (0.4)
(4) Having central type palsy	1.1 (0.4)	1.1 (0.5)	1.1 (0.3)	1 (0.1)
(5) Sublingual varicosities	2.6 (0.9)	2.3 (0.9)	1.7 (0.7)	1.3 (0.5)
(6) Dark purple of palate mucosa	1.7 (0.5)	1.5 (0.5)	1.4 (0.5)	1.2 (0.3)
(7) Blackish red gingiva	1.7 (0.7)	1.4 (0.5)	1.3 (0.6)	1.1 (0.3)
(8) Blackish red lips	2.2 (0.9)	1.9 (0.7)	1.6 (0.8)	1.4 (0.6)
(9) Blackish red tongue	1.9 (0.7)	1.7 (0.6)	1.7 (0.9)	1.3 (0.5)
(10) Ecchymosis of tongue	1.1 (0.3)	1.1 (0.3)	1.4 (0.6)	1.1 (0.3)
(11) Dark coloration of periocular region	2.1 (0.7)	1.7 (0.6)	1.5 (0.6)	1.3 (0.5)
(12) A dark coloration of the face	1.8 (0.7)	1.4 (0.5)	1.4 (0.6)	1.2 (0.4)
(13) Ecchymosis of skin	1.3 (0.6)	1.1 (0.3)	1.3 (0.7)	1.2 (0.5)
(14) Scaly and rough skin	2.1 (1)	1.7 (0.9)	1.4 (0.9)	1.1 (0.5)
(15) Palpable abdominal masses	—	—	—	—
(16) Having painful menstruation or dysmenorrhea	—	—	—	—
(17) Blackish red menstruation blood	—	—	—	—
(18) Accompanied by clotted blood	—	—	—	—
(19) Rough pulse	2.1 (0.8)	1.5 (0.5)	1.8 (0.3)	2 (0.1)
(20) Telangiectasia or angioectasia	1.6 (0.7)	1.3 (0.6)	1.3 (0.7)	1.1 (0.5)
(21) Palmar erythema	1.7 (0.7)	1.5 (0.6)	1.3 (0.4)	1.2 (0.4)
(22) Bruised easily	2.4 (1.2)	1.7 (1)	3 (1.6)	1.8 (1.3)
(23) Tenderness and resistance of navel region	2.6 (1)	1.9 (0.8)	3.2 (1.2)	2 (1)
(24) Tenderness and resistance of ileocecum	2.2 (1)	1.7 (0.8)	3 (1.2)	1.6 (0.7)
(25) Tenderness and resistance of sigmoid colon	2.1 (1)	1.7 (0.7)	2.8 (1.2)	1.7 (0.9)
(26) Tenderness and resistance of hypochondrium	1.9 (0.9)	1.5 (0.7)	1.9 (1.2)	1.2 (0.5)
(27) Lower abdominal pain	1.6 (0.9)	1.2 (0.5)	4.4 (1.1)	4.9 (0.5)
(28) Cheek pain	1.3 (0.6)	1.1 (0.4)	1.4 (0.9)	1.1 (0.5)
(29) Having pain due to sprain of ankle, wrist and spine	1.7 (1)	1.2 (0.4)	1.4 (1)	1.2 (0.6)
(30) Having symptoms (pain, bruise) due to contusion or traffic accident	1.7 (1.2)	1.1 (0.3)	2.8 (1.8)	2.1 (1.6)
(a) Number of traffic accidents	—	—	—	—
(31) Chronic pain in joint/palsies and numbness	2.4 (1.1)	1.8 (0.9)	2.3 (1.4)	1.4 (0.9)
(32) Stabbing pain	2.6 (1.3)	1.7 (1)	2.8 (1.7)	1.3 (0.8)
(33) Pain at night	1.9 (1.1)	1.2 (0.5)	2.4 (1.6)	1.3 (0.8)
(34) Hemorrhoid	1.5 (0.8)	1.4 (0.7)	1.4 (0.9)	1.2 (0.5)
(35) Dark stool	1.6 (0.9)	1.4 (0.7)	1.1 (0.6)	1 (0.2)
(36) Number of operations	—	—	—	—

BSS score	55.7 (7.9)	44.8 (5.3)	57.7 (8.3)	43.7 (5.2)

Mean (SD).

**Table 3:** The sensitivity, specificity, positive predictive value, and negative predictive value for optimal cut-off value of the BSS score.

	Cut-off value of BSS score	Sensitivity	Specificity	PPV	NPV
2013	48	84.8%	78.8%	81.5%	82.4%
2014		86.3%	79.2%	75.5%	88.6%
2013	49	80.8%	83.2%	84.2%	79.7%
2014		84.6%	83.1%	78.7%	87.9%
2013	50	74.4%	86.3%	85.7%	75.3%
2014		81.1%	88.1%	83.5%	86.3%

PPV: positive predictive value; NPV: negative predictive value.
